# A small erythropoietin derived non-hematopoietic peptide reduces cardiac inflammation, attenuates age associated declines in heart function and prolongs healthspan

**DOI:** 10.3389/fcvm.2022.1096887

**Published:** 2023-01-18

**Authors:** Nolan M. Winicki, Alay P. Nanavati, Christopher H. Morrell, Jack M. Moen, Jessie E. Axsom, Melissa Krawczyk, Natalia N. Petrashevskaya, Max G. Beyman, Christopher Ramirez, Irene Alfaras, Sarah J. Mitchell, Magdalena Juhaszova, Daniel R. Riordon, Mingyi Wang, Jing Zhang, Anthony Cerami, Michael Brines, Steven J. Sollott, Rafael de Cabo, Edward G. Lakatta

**Affiliations:** ^1^Laboratory of Cardiovascular Science, Intramural Research Program, National Institute on Aging, Baltimore, MD, United States; ^2^Laboratory of Experimental Gerontology, Intramural Research Program, National Institute on Aging, Baltimore, MD, United States; ^3^Araim Pharmaceuticals, Inc., Tarrytown, NY, United States; ^4^Feinstein Institutes for Medical Research, Northwell Health, Manhasset, NY, United States

**Keywords:** inflammaging, longitudinal study, cardiovascular aging, healthspan, frailty, cardiac inflammation

## Abstract

**Background:**

Aging is associated with increased levels of reactive oxygen species and inflammation that disrupt proteostasis and mitochondrial function and leads to organism-wide frailty later in life. ARA290 (cibinetide), an 11-aa non-hematopoietic peptide sequence within the cardioprotective domain of erythropoietin, mediates tissue protection by reducing inflammation and fibrosis. Age-associated cardiac inflammation is linked to structural and functional changes in the heart, including mitochondrial dysfunction, impaired proteostasis, hypertrophic cardiac remodeling, and contractile dysfunction. Can ARA290 ameliorate these age-associated cardiac changes and the severity of frailty in advanced age?

**Methods:**

We conducted an integrated longitudinal (*n* = 48) and cross-sectional (*n* = 144) 15 months randomized controlled trial in which 18-month-old Fischer 344 x Brown Norway rats were randomly assigned to either receive chronic ARA290 treatment or saline. Serial echocardiography, tail blood pressure and body weight were evaluated repeatedly at 4-month intervals. A frailty index was calculated at the final timepoint (33 months of age). Tissues were harvested at 4-month intervals to define inflammatory markers and left ventricular tissue remodeling. Mitochondrial and myocardial cell health was assessed in isolated left ventricular myocytes. Kaplan–Meier survival curves were established. Mixed ANOVA tests and linear mixed regression analysis were employed to determine the effects of age, treatment, and age-treatment interactions.

**Results:**

Chronic ARA290 treatment mitigated age-related increases in the cardiac non-myocyte to myocyte ratio, infiltrating leukocytes and monocytes, pro-inflammatory cytokines, total NF-κB, and p-NF-κB. Additionally, ARA290 treatment enhanced cardiomyocyte autophagy flux and reduced cellular accumulation of lipofuscin. The cardiomyocyte mitochondrial permeability transition pore response to oxidant stress was desensitized following chronic ARA290 treatment. Concurrently, ARA290 significantly blunted the age-associated elevation in blood pressure and preserved the LV ejection fraction. Finally, ARA290 preserved body weight and significantly reduced other markers of organism-wide frailty at the end of life.

**Conclusion:**

Administration of ARA290 reduces cell and tissue inflammation, mitigates structural and functional changes within the cardiovascular system leading to amelioration of frailty and preserved healthspan.

## Introduction

Chronic inflammation has been linked to the age-associated deterioration of organ structure and function throughout the body. Specifically, “inflammaging” was coined to describe low-grade, chronic, systemic inflammation that accompanies the aging process in the absence of infection, and is a significant risk factor for both morbidity and mortality in older persons (1). Strong evidence from mechanistic studies have shown that inflammaging is a major risk factor for cardiovascular disease (CVD) and many other age-associated adverse health outcomes (2). Previous investigations have shown how the aging rodent manifests abnormalities in left ventricular hypertrophy, fibrosis, and cardiac diastolic dysfunction that recapitulate many of those described in humans (3–5). Specifically, the rodent heart also becomes hypertrophic with age and there is a substantial decline in systolic and diastolic function (4–7). Additionally, the role of oxidative stress, proteostasis, and mitochondrial dysfunction have been shown to influence cardiac and system wide functions in both animal models and humans (8–12). Because aging, per se, is the major risk factor for the development of the most common CVDs (13–15), there is a need for further development of safe interventions that will antagonize the effects of inflammation that underpins age-associated changes in cardiac structure and function.

Erythropoietin (EPO) had been previously investigated as a potential therapeutic for CVD due to its native role as a potent anti-inflammatory cytokine (16). However, the potential therapeutic benefits of direct EPO therapy was outweighed by its primary erythropoietic effects to increase the red blood cell mass and subsequently, the risk of thromboembolic complications (17). Thus began the development of ARA290 (cibinetide), an 11-amino acid peptide derived from the tissue protective domain of erythropoietin, but lacking erythropoietic effects (18). ARA290 mediates tissue protection by reducing inflammatory and fibrotic responses by *via* signaling *via* a heterodimeric receptor composed of EPOR and CD131, the β common cytokine receptor, which also forms receptor complexes with the α receptor subunits specific for GM-CSF, IL-3, and IL-5 (18–20).

The β-common-receptor acts in conjunction with the EPO receptor to form a heterocomplex that is rapidly up-regulated locally following tissue injury (21), initiating a local anti-inflammatory response, inhibition of death signals and anti-apoptosis that aids in the prevention of tissue damage. Chronic treatment with ARA290 reduces systemic inflammation by decreasing susceptibility to diet-induced insulin resistance, neuropathic pain in patients with type 2 diabetes and sarcoidosis associated small nerve loss (22–25). Additionally, ARA290 administration to rats improves survival following myocardial infarction, reduces organ dysfunction in mice during hemorrhagic shock, and suppresses development of atherosclerosis in hyperlipidemic rabbits (26–28).

While administration of ARA290 has shown benefits in multiple clinical disease states, the efficacy of ARA290 treatment upon the chronic inflammation endured as age advances, and subsequent cardiac structural and functional changes that accompany advancing age, have not been established. We hypothesized that chronic ARA290 administration to middle aged rats will reduce cardiac mitochondrial dysfunction, improve autophagy and proteostatsis, and ameliorate cardiac structural remodeling and functional deterioration that are known to occur in rats of advanced age and that signs of frailty will be reduced, thereby improving healthspan.

## Materials and methods

### Study design

All animals were used in compliance within the National Institute of Health Animal Care and Use Committee. The present study is an integrated longitudinal (*n* = 48) and cross-sectional (*n* = 144) controlled trial in which 18-month-old Fischer 344 x Brown Norway rats, were randomly assigned to either receive chronic ARA290 treatment (100 μg/kg) or saline treatment intraperitoneally tri-weekly (Supplementary Figure 1). Longitudinal assessments were evaluated every 4 months starting at baseline and ending following 15 months of ARA290 or saline treatment when rats were 33 months of age. Subsets of the total rat cohort were sacrificed for tissue analysis at baseline and after 4, 12, and 15 months of ARA290 or saline treatment. The criteria for which rats were euthanized was based upon the study approved ACUC protocol maintained by the NIH vivarium veterinarian. The moribund condition is defined in our study as a clinically irreversible condition leading inevitably to death. Specifically, any rat that was found unexpectedly to be moribund, cachectic, or unable to obtain food or water was euthanized. The moribund rats were chosen to be culled at the major data collection endpoints.

#### Longitudinal assessment of cardiovascular structure and function, blood pressure, and body weight

Methods employed for echocardiography, body weight, frailty index, left ventricular (LV) histology, immunohistochemistry, Western blotting, myocyte isolation, and mitochondrial function are described in detail in the Supplementary material. Briefly, repeated echocardiography was performed under light anesthesia as described previously (29). Repeated measures of tail blood pressure (BP) were obtained in conscious animals using the IITC Tail Blood Pressure system (MRBP, IITC, Woodland Hills, CA, USA) as described previously. Repeated measures of all rats were weighed weekly between the hours of 9 a.m. and 12 p.m. on the same day every week. Observations of body weight were made by two observers and recorded by a third. Values were read to the nearest tenth of a gram.

#### Cross-sectional assessment of frailty, LV histology, and western blot analysis

Frailty was assessed using the Howlett and Rockwood non-cardiac clinical frailty index developed for rodents, which includes 31 different parameters (30–32).

Left ventricular Histology was performed on LV samples that were divided and stored into three equal portions along the long axis identified as the apex, middle, and heart base as described previously (33). Morphometrical analyses of myocardial cardiomyocyte, collagen deposition, epicardial coronary arteries was performed using computerized imaging analysis system (Metamorph, University Imaging) by two investigators who were blinded to the treatment protocol based on modified previous descriptions (34). See Supplementary material for further experimental methods.

Western blot analysis was performed as previously described, see Supplementary material for details (35). The complete list of antibodies utilized can be found in Supplementary Table 1.

### Mitochondrial permeability transition, autophagy, and lipofuscin accumulation in single cardiac myocytes

Single ventricular myocytes were isolated and prepared from Fischer 344 x Brown Norway rats by standard enzymatic techniques, as previously described (36).

mPTP-ROS threshold was assessed as described previously, using a method to quantify the ROS susceptibility for the induction of mPTP in individual mitochondria within cardiac myocytes (Supplementary Figure 2; 37).

Autophagic Activity and Lipofuscin Accumulation were assessed *via* images of cardiomyocytes stained for autophagolysosomes and lipofuscin accumulation and analyzed by MetaMorph image analysis software (Molecular Devices), as described previously (Supplementary Figure 2; 38). See Supplementary material for further experimental methods.

### Statistics

All statistical analyses were implemented in R 3.2.3 using RStudio. Data are presented as mean ± standard deviation. The average age trajectory is displayed using a loess smooth curve. Linear mixed effect models were utilized to capture the longitudinal trajectories of all the cardiac parameters reported (lmerTest). Mixed ANOVA models were also used to analyze the data (lmer function). These models account for the repeated measures though a random effect for rat. *Post hoc* comparisons are conducted using the FDR approach to adjust *p*-values for multiple testing. See Supplementary material for detailed account of statistics.

## Results

### Age-associated deterioration in LV structure and function

#### Left ventricular function

**FIGURE 1:**
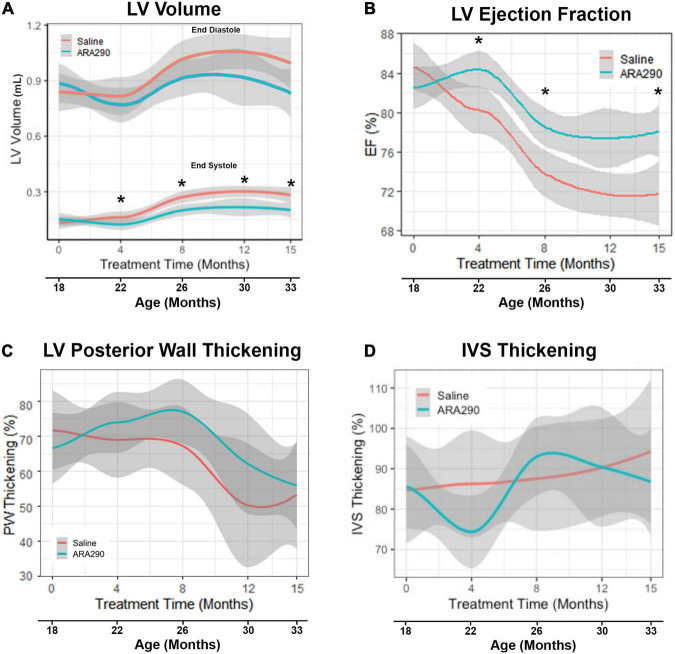
Loess smooth curve (Red = Saline, Blue = ARA290) with 95% confidence intervals (gray) depicting the age-associated changes in left ventricular volume **(A)**, ejection fraction **(B)**, posterior wall thickening percentage **(C)**, and interventricular septum thickening percentage **(D)**. Statistically significant interactions between treatment groups are shown (*P < 0.05).

**FIGURE 2:**
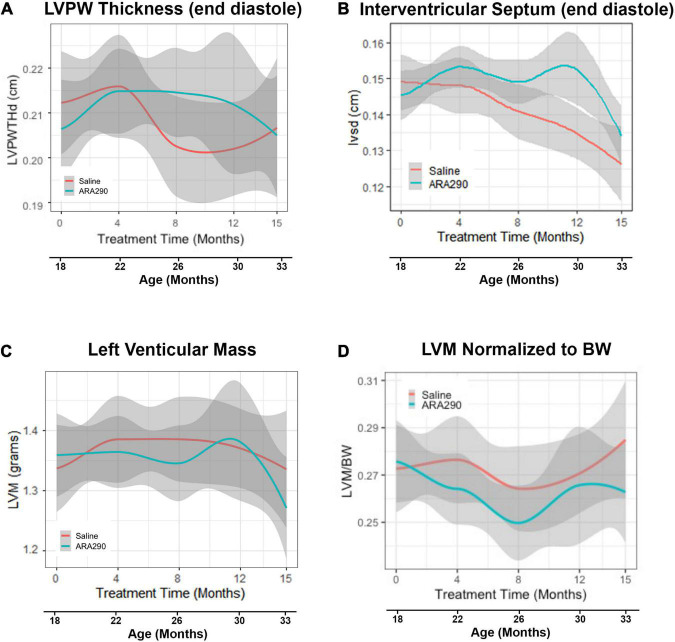
Loess smooth curve (Red = Saline, Blue = ARA290) with 95% confidence intervals (gray) depicting the age-associated changes in left ventricular posterior wall thickness at end diastole **(A)**, interventricular septum posterior wall thickness at end diastole **(B)**, left ventricular mass **(C)**, and left ventricular mass normalized to rat body weight **(D)**. Statistically significant interactions between treatment groups are shown.

Echocardiographic analysis of ARA290 peptide treatment unveiled an ARA290-dependent prevention of age-related deterioration of systolic cardiac function (summarized in Supplementary Tables 2, 3, and selectively illustrated in Figures 1, 2).

In control rats, between 18 and 33 months there was an approximate two-fold increase (*P* < 0.0001) in left ventricular (LV) volume at end-systole (LVES) and an approximate 25% increase (*P* < 0.0001) in LV volume at end-diastole (LVED) (Figure 1A). These results indicate an age-associated loss in LV function, failure to squeeze down at the end of the heartbeat leading to increased volume at the start of the next heartbeat. Stroke volume, the amount of blood pumped during each beat, did not change significantly with age (Supplementary Table 2), but LV ejection fraction (EF), the fraction of blood in the LV at the beginning of the heartbeat that was pumped out during the heartbeat, progressively decreased with advancing age by about 14%, beyond 22 months (*P* < 0.0001) (Figure 1B). Percent LV posterior wall (LVPW) thickening (difference in thickness at end systole minus at end diastole divided by thickness at end diastole) significantly decreased by 30% with age (*P* < 0.01), indicating that the effectiveness of the contraction of heart muscle became less as age advanced. However, the interventricular septum thickness percentage did not significantly change (Figures 1C, D).

#### Left ventricular structure

Although the LV posterior wall thickness *at end diastole* (LVPWTd) did not change significantly with advancing age (Figure 2A), the interventricular septal thickness at end diastole (IVSTd) became significantly reduced (by around 16%) as age increased (*P* < 0.01) (Figure 2B). LV mass (LVM, calculated from the wall thickness and diameter) did not change significantly across the broad age range from 18 to 33 months, but the LVM normalized to body weight significantly *decreased* between 18 and 26 months, and significantly increased thereafter (Figures 2C, D), due to the progressive reduction in body weight in late life.

#### ARA290 treatment mitigated age-associated LV functional decline

ARA290 treatment significantly mitigated the age-associated increase in LVES (by approximately 75%), with treated rats maintaining a significantly lower LVES starting at 4 months of treatment (22 months of age) and continuing until the end of the experiment (33 months of age) (*P* < 0.05) (Figure 1A). Although ARA290 treatment did not significantly impact the age-associated increase on LVED, it significantly blunted the age-associated decline in EF (by almost a half due to its effect to reduce LVES), beginning at 4 months of treatment and continuing throughout the experiment (*P* < 0.0001). ARA290 treatment did not have a significant impact on the age-associated increase in percentage LVPW thickening.

ARA290 treatment did not significantly affect the age-associated LV structural changes, represented by the increase in the LVPWTd and IVSTd and did not affect LVM or myocardial wall thinning (Figure 2).

#### ARA290 treatment reduced tail blood pressure elevation with age

**FIGURE 3:**
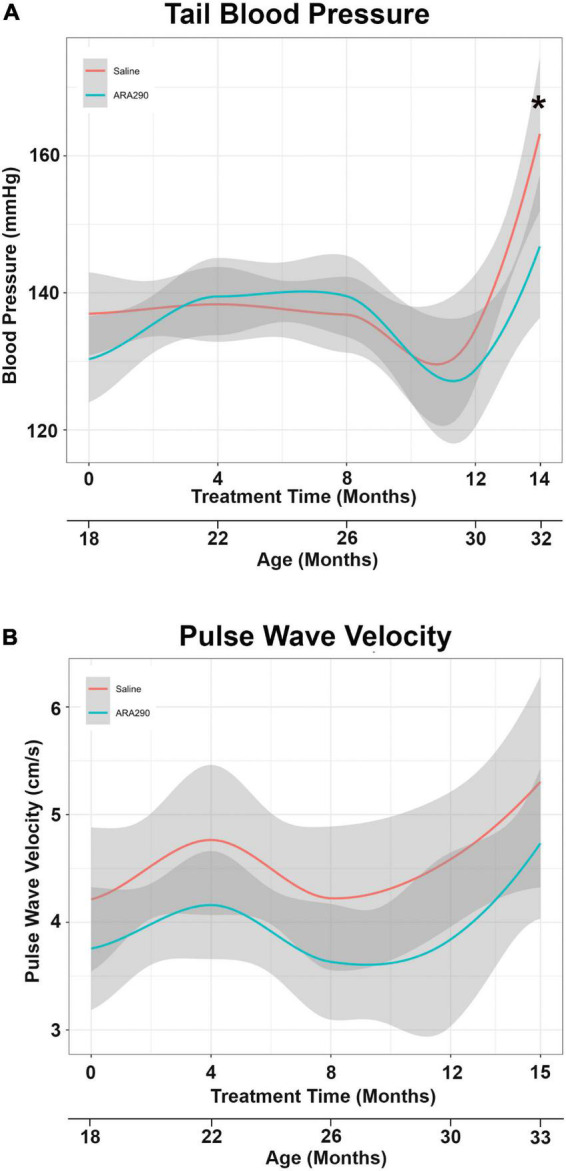
Loess smooth curve (Red = Saline, Blue = ARA290) with 95% confidence intervals (gray) depicting the age-associated changes in tail blood pressure **(A)** and pulse wave velocity **(B)**. The final tail blood pressure reading was collected 1 month prior to the experiment endpoint. Statistically significant interactions between treatment groups are shown (*P < 0.05).

Pulse wave velocity (PWV) and tail blood pressure (Figures 3A, B) both rose significantly over the lifespan (by 26%, *P* < 0.05 and 17%, *P* < 0.01, respectively). ARA290 mitigated the abrupt elevation in tail BP (by more than half) that was observed beyond 12 months of treatment (*P* < 0.008), whilst having no significant effect on the age-associated increase PWV (Figures 3A, B).

#### Late life body weight decline was blunted by ARA290 treatment

**FIGURE 4:**
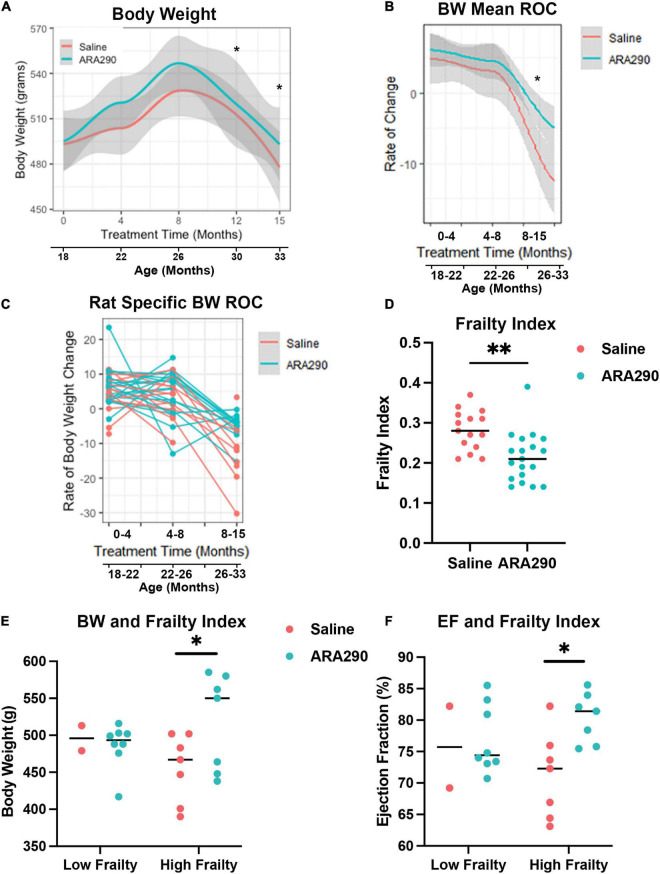
Loess smooth curve (Red = Saline, Blue = ARA290) with 95% confidence intervals (gray) depicting the age-associated changes in body weight **(A)**, mean rate of change in body weight **(B)**, and rat-specific rate of change in body weight **(C)**. The frailty index for all rats at 33 months of age by treatment group **(D)**. Rat body weight and frailty category (frailty index score > 0.21) by treatment group **(E)**. Ejection fractions of rats by frailty category and treatment group **(F)**. Statistically significant interactions between treatment groups are shown (*P < 0.05, ^**^P < 0.01).

Body weight increased in saline treated rats to a maximum at 28 months of age but then steadily decreased until the end of the experiment (33 months of age). Treatment with ARA290 significantly blunted late life weight loss, starting at 26 months of age, with rats treated showing significantly higher body weights at the last two timepoints (*P* < 0.01 and *P* < 0.01) (Figure 4A). ARA290 treatment also blunted the mean rate and rat-specific rates of body weight decline in late life (Figures 4B, C).

#### Systemic frailty in late life was ameliorated by ARA290 treatment

Rats treated with ARA290 scored significantly lower on the overall frailty index at 33 months of age (*P* < 0.001) indicating that in advanced age ARA290 treated rats were significantly less frail than the saline treated rats (Figure 4D).

Further investigation revealed the difference in frailty score is driven by measures of the integument, musculoskeletal, vestibulocochlear, and auditory systems. Specifically, ARA290 rats scored lower than the saline rats on the frailty index by 0.20 for coat condition (*P* < 0.01), 0.31 for hunched posture (*P* < 0.02), 0.32 for body condition (*P* < 0.006), 0.11 for hearing loss (*P* < 0.04), and 0.18 for piloerection (*P* < 0.04). Additionally, there are borderline differences where ARA290 rats scored lower on the frailty index by 0.12 for loss of fur color (*P* < 0.05), 0.07 for loss of whiskers (*P* < 0.08), and 0.12 for unusual sounds (*P* < 0.05). Importantly, at 33 months of age, the rats identified as having high frailty (frailty index score > 0.21), those in the ARA290 group exhibited significantly higher body weights and ejection fractions than high frailty rats in the saline group (Figures 4E, F).

Thus, treatment with ARA290 beginning at 18 months of age reduced the increase in blood pressure, severity of frailty near the end of life and reduced the progressive age-associated deterioration of systolic LV function beyond 22 months of age.

#### LV cardiac myocyte size, non-cardiac myocytes, and collagen deposition increases with age

**FIGURE 5:**
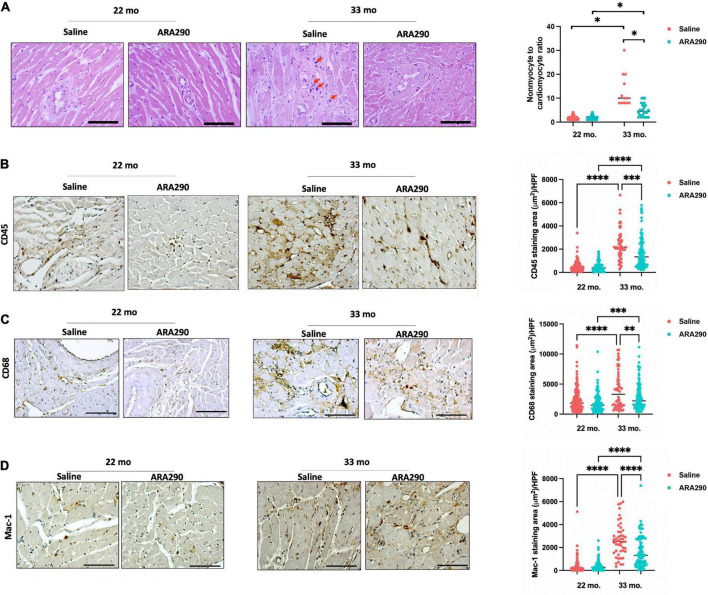
Histomorphometric analysis at 22 (Saline *n* = 10; ARA290 *n* = 11) and 33 (Saline *n* = 4; ARA290 *n* = 8) months of age. Ratio of cardiac non-myocytes to myocytes within the myocardium and interstitial giant cells (red arrow) at 22 and 33 months of age **(A)**. Staining of myocardium with CD45, a pan-leukocyte marker **(B)**, CD68, a marker of inflammation associated with monocytes/macrophages **(C)**, and Mac-1, an integrin that facilitates recruitment of leukocytes **(D)** at 22 and 33 months of age. Statistically significant interactions between treatment groups are shown (*P < 0.05, ^**^P < 0.01, ^***^P < 0.001, ^****^P < 0.0001).

Morphometrical analyses of histologic sections of the myocardium indicated that advanced age was associated with an increased cardiac myocyte cross-sectional area and increased interstitial and peri-intra-epi-coronary artery fibrosis (Supplementary Figures 3A, B). Collagen 5 and 8, thought to increase due to response from vascular injury (39), both increased linearly with aging between 18 and 33 months of age by Western blot (Supplementary Figures 3C, D) (*P* < 0.001 and *P* < 0.001, respectively). Total cardiac myocyte density significantly decreased with age (Supplementary Figure 4A), and the ratio of cardiac non-myocytes to myocytes, an index of non-myocyte proliferation and proinflammation within the myocardium, increased with age (Figure 5A). These findings suggest that age significantly impacts remodeling of the cardiac muscle and non-muscle compartments within the myocardium. Mast cells were one type of cell that comprised the non-myocyte preparation. Interstitial giant cells, potentially macrophages, were an additional non-myocyte cell type (Figure 5A) observed in old age (33 months). Evidence of inflammatory cell infiltration *via* CD45, a pan-leukocyte marker, and CD68, a histochemical/cytochemical marker of inflammation associated with monocytes/macrophages, was observed to increase with age (Figure 5B). Levels of CD45 and CD68 displayed a 4.8- and 1.7-fold age-associated increase (*P* < 0.001 and *P* < 0.001, respectively). Mac-1, an integrin that facilitates recruitment of leukocytes to the site of injury and serves as a molecular link between inflammation and thrombosis, demonstrated a 4.7-fold age-associated increase (Figures 5C, D).

#### ARA290 diminished the age-associated increase in myocardial inflammatory cell infiltration

Although ARA290 treatment did not significantly affect the age-associated increase in myocyte cross-sectional area, myocardial fibrotic score, or increase in collagen 5 and 8, it mitigated the age-associated increase in the ratio of cardiac non-myocytes to myocytes (Figure 5A) and blunted the age-associated rise in inflammatory cells shown by significantly lower staining areas of CD45, CD38, and Mac-1 (Figures 5B–D). Interestingly, ARA290 treatment promoted the positive remodeling (dilation) of coronary arteries (Supplementary Figure 4B).

#### Age-associated increase in myocardial pro-inflammatory cytokines was reduced by ARA290 treatment

**FIGURE 6:**
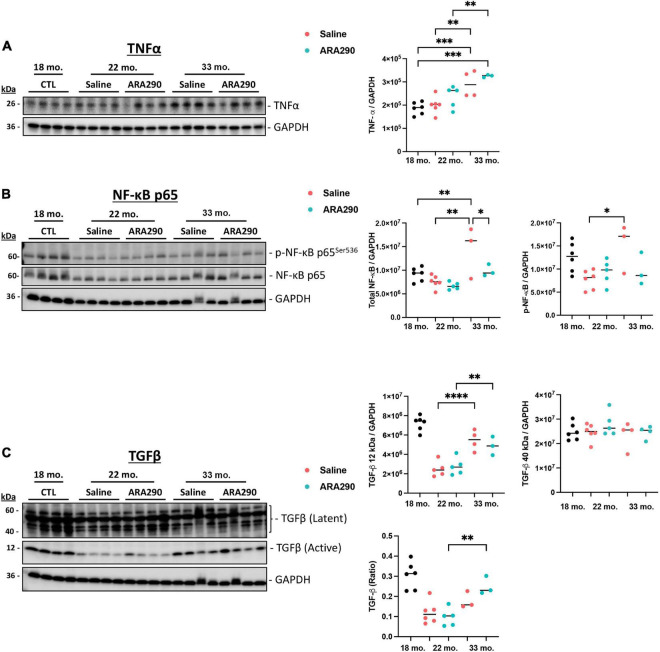
Western blot analysis depicting expression levels of TNF-α **(A)**, p-NF-κB and total NF-κB **(B)**, and TGF-β (12 and 40 kDa) **(C)** relative to GAPDH at 18 (*n* = 6), 22 (Saline *n* = 6; ARA290 *n* = 5), and 33 (Saline *n* = 4; ARA290 *n* = 4) months of age. Statistically significant interactions between treatment groups are shown (*P < 0.05, ^**^P < 0.01, ^***^P < 0.001, ^****^P < 0.0001).

Compared to baseline at 18 months of age, levels of pro-inflammatory markers in the heart were significantly elevated at the end of the experiment (33 months of age) TNF-α (by 45%, *P* < 0.001), p-NF-κB and total NF-κB (by 95 and 89% respectively, *P* < 0.001 and *P* < 0.05, respectively) (Figures 6A, B). The latent form of TGF-β (40 kDa) did not change in advanced age but the active form of the pleiotropic cytokine, TGF-β (12 kDa), progressively doubled between 22 and 33 months of age (*P* < 0.001) as did the ratio of active TGF-β (12 kDa) to the latent form of TGF- β (40 kDa) (*P* < 0.001) (Figure 6C).

Thus, ARA290 treatment significantly blunted the age-associated rise in both p-NF-κB and total NF-κB levels (*P* < 0.05 and *P* < 0.001) but minimally impacted levels of TNF-α (Figures 6A, B). The ratio of TGF-β (40 kDa to 12 kDa) surprisingly increased significantly throughout life only in the ARA290 treated group (*P* < 0.01) (Figure 6C).

#### Mitochondrial permeability transition, autophagy, and lipofuscin accumulation

##### ARA290 elevated the mitochondrial permeability transition pore (mPTP) threshold

**FIGURE 7:**
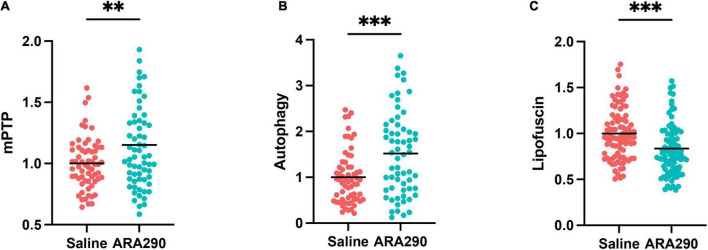
The mitochondrial permeability transition pore (mPTP) opening thresholds **(A)**, cardiomyocyte autophagy flux **(B)**, and accumulation of lipofuscin **(C)** in left ventricular cardiomyocytes isolated from 32-month-old rats. Statistically significant interactions between treatment groups are shown (^**^P < 0.01, ^***^P < 0.001).

Left ventricular cardiomyocytes (CM) isolated from 32-month-old rats that have been continually treated with ARA290 from 18 months of age demonstrated higher resistance to controlled oxidative stress-mediated mitochondrial permeability transition pore (mPTP) opening, as reflected in significantly higher mPTP-ROS thresholds than those of saline treated rats (see methods in Supplementary material), indicating relative constitutive desensitization of the CM mPTP to oxidant stress (*P* < 0.01) (Figure 7A).

##### ARA290 enhanced autophagic flux

The CM autophagy flux, which in cardiac myocytes is dominated by mitochondrial protein turnover, and assessed by the presence of autophagolysosomes was significantly enhanced in ARA290 treated rats compared to those treated with saline, indicating that treatment has a positive effect to protect mitochondrial proteostasis in advanced age (*P* < 0.001) (Figure 7B).

##### Age-associated lipofuscin accumulation was reduced by ARA290 treatment

The accumulation of undegradable cellular “wear-and-tear” byproduct, lipofuscin (Figure 7C), widely recognized to increase with aging, and reflects an impairment of protein recycling mechanisms, was reduced in ARA290 treated vs. saline treated cardiomyocytes (*P* < 0.001).

Collectively, the data in Figure 7 indicates that chronic treatment with ARA290 confers increased cardiomyocyte mitochondrial fitness and cellular stress resistance compared to that in age-matched 32 months old) saline treated controls.

#### Survival analysis

The *median* lifespans and overall survival were not significantly different between the ARA290 and saline treated rats (18–33 months old) (*P* = 0.182) (Supplementary Figure 5).

## Discussion

Cardiac remodeling and functional deterioration are known to accompany advancing age in humans and animal models and are the major risk factors for the development of CVD in older persons. Chronic inflammation, mediated by cytokines TNF-α, IL-6, and NF-κB and increased reactive oxygen species, a reduction in proteostasis and mitochondrial health, are known to increase with advancing age and are linked to the development of progressive LV structural and functional changes observed in older persons (40–42). Although previous studies had demonstrated that EPO reduces inflammation, cytokine production, fibrosis, and oxidative damage in the myocardium, chronic administration of EPO led to an increase in red blood cell mass and subsequent elevated stroke risk. ARA290 was subsequently designed to antagonize the production of inflammatory cytokines and provide cardioprotective benefits without erythropoietic effects (28).

The present study documented: (1) longitudinally defined selected aspects of cardiovascular structural and functional deterioration, blood pressure elevation and aortic stiffening with age, in the context of increased heart inflammation, paralleling initial body weight gain prior to mid-late-life and reduction in body weight beyond mid-life to near end-life in Fischer 344 x Norway Brown rats; (2) characterized age-associated changes in LV histomorphology; (3) established the status of LV mitochondrial health and proteostasis in late-life; (4) characterized selected aspects of LV inflammation.

Longitudinal age-associated changes in cardiovascular structure and function in Fischer 344 x Norway Brown rats included: (1) dilation of the LV diameter in systole and diastole; (2) reduction in LV wall thickness; (3) reduction in diastolic LV wall thickness; (4) reduced percentage LV wall thickening from systole to diastole; (5) reduced LV fractional shortening; (6) a reduction in ejection fraction; (7) increased blood pressure and arterial stiffening in late life. Age-associated changes in histomorphology included an increase in LV myocyte size, increase in non-myocyte to cardiac myocyte ratio, increase in infiltrating cardiac leukocytes and monocytes, increased fibrosis, an increase in LV cytokine levels, and a reduction in proteostasis leading to accumulation of insoluble protein degradation byproducts, i.e., lipofuscin.

Chronic ARA290 treatment reduced the age-associated deterioration in systolic cardiac heart function, reduced signs of LV tissue inflammation, including a reduction in the non-cardiomyocyte to cardiomyocyte ratio that increased with age, ameliorated the age-associated increase in inflammatory leukocytes and monocytes, blunted the age-associated rise in p-NF-κB and total NF-κB, improved mitochondrial proteostasis, blunted late life weight loss and, reduced systemwide frailty in advancing age.

Specific major cardiac benefits of ARA290 were to ameliorate the deterioration of LV systolic function, as indicated by a reduction of the age-associated increase in LV end systolic volume, a reduction in the age-associated deterioration of LV ejection fraction and fractional shortening that progressively decreased over the lifespan (Figure 1). A mitigation of LV functional deterioration and myocardial infarct expansion by ARA290 treatment in a model of myocardial infarction has previously been observed (28).

Chronic administration of ARA290 also reduced the age-associated ratio increase of non-myocyte to myocytes, blunted the age-associated increase in inflammatory leukocytes and monocytes and dynamically remodeled epicardial coronary arteries to increase lumen area (Figure 5; Supplementary Figures 3, 4). Cardiac non-myocytes, typically endothelial cells, resident mesenchymal cells and leukocytes are critical for maintaining homeostasis of the heart (43). However, an increased ratio of cardiac non-myocytes to myocytes has been associated with diastolic dysfunction and pathologic cardiac hypertrophy (44, 45). Specifically, established mediators of cardiac hypertrophy such as atrial natriuretic peptide, brain natriuretic peptide and angiotensin-II have shown to increase the ratio of non-myocyte to myocytes (46, 47). Aging has been shown to cause rarefaction of intramyocardial coronary arterioles, potentially increasing coronary resistance (48). Although the epicardial coronary artery lumen area did not vary with age, lumen area increased in rats receiving ARA290 treatment (Supplementary Figure 4). Other studies have shown the beneficial effects of EPO on coronary and pulmonary vasculature, potentially through a mechanism involving nitric oxide (49, 50).

ARA290 treated rats also showed increased resistance to oxidative stress and enhanced mitochondrial fitness in late life (Figure 7). Cardiomyocytes from these ARA290 treated rats showed higher mPTP-ROS thresholds, autophagy flux and reduced buildup of lipofuscin. Multiple agents and conditions have been observed to induce pore opening in isolated mitochondria, most notably high Ca^2+^ loading and treatments that cause oxidative stress (51). Our findings corroborate previous studies that identified the protective role of ARA290 to moderate the cytotoxicity, genotoxicity, and oxidative stress induced by the chemotherapeutic agent doxorubicin (52); to reduce radiation induced cell apoptosis, oxidative stress, and inflammation when administered as a nanoreactor (53); and to improve glucose oxidation rate, ATP production and enhanced glucose-stimulated insulin secretion in experimental models of diabetes (53).

Cardiac anti-inflammatory effects of chronic ARA290 treatment were manifested as blunting myocardial levels of the major pro-inflammatory cytokines, p-NF-κB, and NF-κB, that rose consistently with age in the saline treated rats (Figure 6). Interestingly, ARA290 did not significantly reduce the age-associated rise in TNF- α, suggesting that there may be an alternative pathway in which ARA290 (or EPO), interacts with NF-κB because TNF-α is a known potent activator of the NF-κB pathway (54). This idea is consistent with a previous study that showed ARA290 treatment decreased the binding affinity of NF-κB to DNA which reduced the mRNA expression of downstream mediators (55). Previous studies have proposed the role of angiotensin II (ATII) as a pro-inflammatory chemokine acting to activate NF-κB, which may contribute to the effects observed in this study (56). Finally, the levels of the pleiotropic cytokine TGF-β increased with age and the ratio of the larger (40 kDa) to smaller (12 kDa) epitope only increased in the ARA290 treated group. TGF-β has been established as the master regulator of fibrosis (57) and also an antagonist of nitric oxide production, which is implicated in the final common pathway of neuropathic pain (58). This has been evaluated in patients with sarcoidosis (59).

Beyond having beneficial effects on LV function, LV tissue inflammation and mitochondrial health, ARA290 reduced a systemwide frailty index in rats of advanced age (Figure 4). Frailty is a state of being highly vulnerable to adverse health outcomes characterized by functional decline in multiple organ systems. Previous studies have shown that LV structure and function deterioration that occur with aging are more highly correlated with the frailty index employed in our study than with chronologic age (60). As such, we found that through evaluation of 31 health-related variables, treatment with ARA290 improved the healthspan in late life. There were similar impacts of ARA290 in body weight (Figure 4). ARA290 treated rats achieved an average peak body weight at an older age than those treated with saline. When expressed as percentage of maximal lifespan in saline or ARA290 treated rats, beyond 80% of the maximum lifespan ARA290 treatment conferred higher maximal body weight, and a slower late-life decline. These results suggest that that the reduction in systemic inflammation, improvement of LV systolic function and retention of late life body weight induced by ARA290 treatment are linked to its effect to attenuate frailty and preserve healthspan in rats of advanced age.

## Conclusion

Treatment of near middle-aged rats with ARA290 attenuated the age-associated decline in cardiac systolic function that presents beyond middle age, reduced frailty and improved late life healthspan in advanced age. Potential mechanisms that underly the effects of ARA290 to reduce the age-associated deterioration of cardiac function include the impact of ARA290 on reduction of cardiac tissue inflammation, on increased resistance to oxidative stress, on preserved protein quality control and mitochondrial function in cardiac myocytes. Systemic effects of ARA290 to preserve overall healthspan in late life are manifested in its ability to attenuate late-life body weight loss in the context of ameliorating body-wide frailty.

## Data availability statement

The raw data supporting the conclusions of this article will be made available by the authors, without undue reservation.

## Ethics statement

This animal study was reviewed and approved by National Institute of Health Animal Care and Use Committee.

## Author contributions

EL, RC, MB, AC, and NW: conceptualization. EL, RC, MB, AC, NW, MW, DR, and CM: methodology. NW, MW, DR, CM, AN, JM, JA, MK, NP, MGB, CR, IA, SM, and MJ: investigation. EL and RC: funding acquisition and supervision. EL, RC, MB, AC, NW, MW, and CM: writing—original draft. EL, RC, MB, AC, NW, MW, DR, CM, AN, JM, JA, MK, NP, MGB, CR, IA, SM, and MJ: writing—review and editing. All authors contributed to the article and approved the submitted version.
