# Supplementary material for: A small erythropoietin derived non-hematopoietic peptide reduces cardiac inflammation, attenuates age associated declines in heart function and prolongs healthspan
Source: Front Cardiovasc Med. 2023 Jan 18;9:1096887. doi: 10.3389/fcvm.2022.1096887 (PMC9889362; doi:10.3389/fcvm.2022.1096887)
Supplement: Supplementary file 1 [file Data_Sheet_1.docx]

**SUPPLEMENTAL MATERIAL**

**METHODS**

***Statistics***

For many ECHO time-series parameters, the trajectory over the age span is highly non-linear, exhibiting sharp changes around 4 and 8 months of treatment, 22 and 26 months of age. Consequently, when needed, the trajectories are modeled in three parts: treatment times 0 – 4 months, 4 – 8 months and 8 to 15 months. To accommodate the curvature in the trajectories within each of these age-spans, a quadratic model in age is adopted, when needed. In this case, the full LME model becomes:


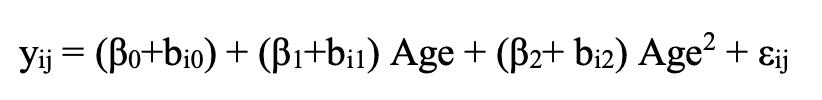


LME models contain both fixed and random terms. The β’s are the regression parameters for the fixed-effects variables, while b_i0_, b_i1_, and b_i2_ are the corresponding random effects. ε_i_ is the usual error term. The fixed-effects terms of LME models are the usual regression-type explanatory variables and their parameter estimates provide estimates of average rates of change in the response variable for changes in the associated variable. However, the random effects terms of LME models allow for variability in initial value and trajectories among the rats. To obtain rat-specific rates of change, age must now be considered as a numerical variable, and the trajectory over the age span must be appropriately modeled. The random intercept effect (b_i0_) allows for variability in individual rat intercepts or starting points, while the additional random effects, b_i1_ and b_i2_, allow for the linear and quadratic terms to vary among the animals. In the full model, the rate of change in the response for individual rat i is the derivative of the model function with respect to age and is given by:


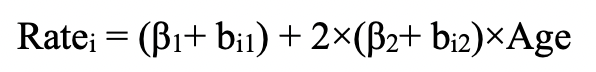


where the β’s and bi’s are replaced by the estimates obtained from the data. When terms are eliminated from the model, the appropriate remaining terms from the final model are used to obtain the rat-specific rates of change. For many variables, the data could not accommodate the random effect for Age2. Consequently, in these cases, this random effect was removed from the model. When comparing continuous data via mixed ANOVAs, all p-values were adjusted for multiple testing. Survival analysis was computed using a logrank test on the Kaplan-Meier survival plot (survminer).

***Echocardiography***

Echocardiograms and electrocardiograms were recorded at the start of the study and at 4, 8, 12 and 15 months into the study. Cardiac function and morphology were assessed with echocardiography (Sonos 5500, 12MHz probe) and was performed under light anesthesia (2-2.5% isoflurane in oxygen). Heart images were obtained in 2-dimensional mode in the parasternal short axis views at midpapillary level. Measurements of LV intraventricular septum (IVS), LV internal diameters (LVID), and posterior wall thickness (LVPW) were made in systole and diastole from M-mode view. Ejection fraction was calculated using the Teichholz formula of: ((7/(2.4+ LVIDd))(LVIDd^3^). LV percent fractional shortening was calculated as: fractional shortening = [(LVIDd-LVIDs)/LVIDd]*100%. Heart mass was calculated from the M-mode measurements of IVS, LVID, and LVPW in diastole using cuboid method. IVS and LV posterior wall thickening from diastole to systole were calculated using (IVSs-IVSd)/IVSs)*100, and (PWs-PWd)/PWd*100, respectively.

For pulse wave velocity (PWV) measurements, ECG leads were placed on 2 front legs and 1 rear leg, and ECG tracing was recorded. Aortic PWV was measured by the transit time method of doppler signal at the ascending aorta and the abdominal aorta. The distance (d) between 2 points was measured. The time at the ascending aorta (t1) and at the abdominal aorta (t2) was defined as the time from the peak of the ECG P wave to the foot of the velocity upstroke. The transit time (Dt) of the flow wave from the ascending aorta to the lower abdominal aorta was determined as the time difference between 2 measurements (t2-t1). Pulse wave velocity (PWV) was calculated as D/dt (m/s).

***Tail Blood Pressure***

Tail blood pressure (BP) was performed in conscious animals using the IITC Tail Blood Pressure system (MRBP, IITC, Woodland Hills, CA). The software was calibrated according to manufacturer instructions. Briefly, animals were trained three times over three days before official measurements were recorded. Animals were preheated for 15 minutes at 32°C and then transferred to an acrylic snap holder supplied by IITC. The platform on which they were held was warmed to 32°C by warm air. A combined occlusion and sensor cuff was placed on the base of the tail. Blood flow to the tail was occluded up to 250 mmHg and released over thirty seconds. Systolic BP was identified as the point of return of regular pulses.

***LV Histology***

LV samples were divided and stored into three equal portions along the long axis identified as the apex, middle, and heart base. ^33^ Fresh slices of left ventricular middle wall were snap frozen in liquid nitrogen for subsequent protein analysis. Adjacent sections (~1.5-2.0 mm in thickness) of the middle wall were fixed in 4% paraformaldehyde for 24 hours and embedded in paraffin for subsequent histological and morphological analyses. Hematoxylin and eosin, picrosirius red, silver (reticulum stain kit, American MasterTech Scientific Inc.), and Masson's trichrome stains (American MasterTech Scientific Inc.) were performed per the manufacturer's instruction.

***Myocardial Immunostaining***

Immunohistochemistry was performed according to the biotin-streptavidin kit instructions (Zymed Laboratories, Inc) and modified methods described previously.^61^ Antigen was recovered in citrate buffer at pH 6.0 (Zymed Laboratories, Inc) for 1-3 minutes in a microwave oven. The slides were incubated overnight at 4°C with primary antibodies. Leukocytes in the myocardium were identified by staining with an antibody against CD45 (Code IS751, leucocyte common antigen clones 2B11 + PD726, Dako), macrophages by staining with an anti-CD68 antibody (Code IS613, Clone PG-M1 Dako), and T & B cells by anti Mac-1 antibody (Code R0841 CD11b, C3bi RecptorRPE, clone 2LPM 19c Dako).^62, 63^

***Morphometrical Analyses of Myocardial Cardiomyocyte, Collagen Deposition, Epicardial Coronary arteries***

In brief, cardiomyocytes were measured in the anterior, middle, posterior regions of left free ventricular wall at the mid-level of ventricle under the cross-sectional view. Cross-sectioned cardiomyocytes with a centrally located nucleus were considered. Twenty to fifty cardiomyocytes from 3 independent fields of images taken at X400 magnification, in left ventricular free wall at the mid ventricular level, using reticular silver stain were measured using the MCID drawing tool to trace cardiomyocyte cross-sectional area. Four to eleven hearts per group were measured. Myocardial interstitial collagen density was measured in the anterior, middle and posterior regions of left free ventricular wall at the mid-level of ventricle under the cross-sectional view. Individual myocardial interstitial collagen density (blue color) under a high-power field was measured in each animal with 10-20 fields from an image taken at X400 magnification and was expressed as the proportion of stained area to target area (density), using Masson’s trichrome stain. Individual epicardial peri-intra-coronary collagen density (blue color) was measured in each animal with 2-3 arterioles from an image taken at X400 magnification and was expressed as the proportion of stained area to the corresponding coronary luminal area, using Masson’s trichrome stain. Individual ratio of non-myocyte to myocyte was determined in each animal in the anterior, middle and posterior regions of left free ventricular wall at the mid-level of ventricle under the cross-sectional images taken at X400 magnification, using a HE stain view. The number of nonmyocytes and myocyte nuclei were counted from small non-myocytes versus the much larger cardiomyocytes. Note: this area count method excluded myocardial arteriolar (perivascular) fibrosis, but included myocardial fibrosis (interstitial fibrosis, also known as a reactive myocardial fibrosis).

Individual epicardial coronary wall thickness index was measured in each animal with 2-3 arterioles from an image taken at X400 magnification and was expressed as the proportion of arterial wall area to the corresponding coronary luminal area, using Masson’s trichrome stain. Semi-quantification of histochemical staining fibrotic score was based on the extent of collagen deposition in a high-power field in each animal with 4-6 fields, using Masson’s trichrome stain: Score 0: no or light (scattered); Score 1: banded or scatter collagen < 10; Score 3: clustered or diffused collagen deposits ≥ 10 and < 25%; Score 4: clustered or diffused collagen deposits ≥ 25%.

***Western Blot Analysis***

Snap-frozen heart tissue was pulverized using a Cellcrusher (Cellcrusher, Cork, Ireland) cooled to liquid nitrogen temperature, then homogenized and lysed in ice cold RIPA buffer (Thermo Fisher Scientific, Waltham, MA): 25 mM Tris-HCl (pH 7.6), 150 mM NaCl, 1% NP-40, 1% sodium deoxycholate, 0.1% SDS) supplemented with Halt protease inhibitor cocktail (Thermo Fisher Scientific), Halt phosphatase inhibitor cocktail (Thermo Fisher Scientific) and 1 mM phenylmethyl sulfonyl fluoride, using a Precellys homogenizer (Bertin Instruments, Rockville, MD) with tissue homogenization kit CKMix (Bertin Instruments) at 4 °C. Extracts were then centrifuged at 10,000×g for 10 min at 4 °C. Insoluble proteins were extracted from the pellet using a pellet extraction buffer (PEB) from the ThermoFisher Scientific Subcellular Protein Fractionation Kit for Tissues according to the manufacturer’s protocol. Protein concentrations of the soluble and insoluble fractions were determined using the Bicinchoninic Acid (BCA) Assay (Thermo Fisher Scientific). Samples were denatured in Laemmli sample buffer (BioRad Laboratories) containing 355 mM 2-mercaptoethanol at either 37^o^C for 10 minutes (for Collagen detection) or 70^o^C for 10 minutes, and proteins (10-50 μg/lane) resolved on 4-20% Criterion™ TGX Stain Free™ gels (Bio-Rad Laboratories, Hercules, CA) by SDS/PAGE. Gels were then exposed to UV transillumination for 2.5 minutes to induce crosslinking of Stain Free™ gel trihalo compound with protein tryptophan residues. Proteins were then transferred to low fluorescence polyvinylidene difluoride (LF-PVDF) membranes (BioRad Laboratories) using an electrophoretic transfer cell (Mini Trans-Blot, Bio-Rad). Membrane total protein was visualized using an Amersham Imager 600 (AI600) (GE Healthcare Life Sciences) with UV transillumination to induce and capture fluorescence signal. Blocked membranes (5% milk/tris-buffered saline with Tween-20, TBST) were incubated with the following primary antibodies: anti-Col1A1 (1310-01) from Southern Biotech (Birmingham, AL) at a 1:1,000 working concentration; anti-TGFβ1 (3709) at 1:1,000, anti-NF-kB p65 (8242) at 1:1,000, anti-GAPDH (2118) at 1:5000, and anti-Pan Actin (8456) at 1:2,000 from Cell Signaling Technology (Danvers, MA); anti-Col3a1 (PA5-27828) at 1:1,000 and anti-p-NF-kB p65 Ser536 (44-711G) at 1:1000, and anti-TNFα (ab205587) at 1:1,000 from Abcam (Cambridge, United Kingdom). Primary antibodies were then detected using horseradish peroxidase (HRP) conjugated antibody (ThermoFisher Scientific) at 1:10,000. Bands were visualized using Pierce SuperSignal™ West Pico Plus ECL substrate kits (ThermoFisher Scientific), the signal captured using an Amersham Imager 600 (AI600) (GE Healthcare Life Sciences) and quantified using ImageQuant TL software (GE Healthcare Life Sciences). GAPDH (soluble fractions) and Actin (insoluble fractions) were used for normalization.

***Left Ventricular Myocyte Isolation***

Briefly, Fischer 344 x Brown Norway rats were anesthetized with sodium pentobarbital, and the hearts were rapidly excised and perfused with 40 mL of Ca^2+-^free bicarbonate buffer gassed with 95% O_2_ to 5% CO_2_ at 37°C. The buffer had the following composition: 116.4 mmol/L NaCl, 5.4 mmol/L KCl, 1.2 mmol/L MgSO_4_ 1.2 mmol/L NaH_2_PO_4_, 5.6 mmol/L glucose, and 26.2 mmol/L NaHCO_3_, pH 7.4. The hearts were continuously perfused with bicarbonate buffer containing 0.1% collagenase type B, 0.04 mg/mL protease XVI, and 0.1% bovine serum albumin type V for 4 min, and 50 µmol/L Ca^2+^ was added. After 10 min of perfusion, the left ventricle was minced and incubated for 10 min at 37°C in bicarbonate buffer containing 100 µmol/L Ca^2+.^ Myocytes then were resuspended in HEPES buffer with gradually increasing Ca^2+^ concentration up to 1 mmol/L and kept at room temperature until use. The HEPES buffer had the following composition: 137 mmol/L NaCl, 4.9 mmol/L KCl, 1.2 mmol/L MgSO_4_ 1.2 mmol/L NaH_2_PO_4_ 15 mmol/L glucose, 20 mmol/L HEPES, and 1.0 mmol/L CaCl_2_ (adjusted pH to 7.4). The isolated cardiac myocyte viability was typically 70% to 80%.

***mPTP-ROS threshold***

Briefly, myocytes were loaded with 125 nmol/L tetramethylrhodamine, methyl ester (TMRM) for at least 1 h at room temperature. The cells were analyzed between 2- and 8-hours post-isolation and were taken from N = 6 rats. Then, myocytes were exposed in vitro to conditions that mimic oxidative stress by repetitive laser scanning of a selected row of ~25 mitochondria arrayed along myofibrils (**Supplemental Figure 2**). This results in incremental, additive exposure of only the laser exposed area to the photodynamic production of ROS and consequent mPTP induction. Line scan images of this mitochondrial raw were recorded at 2 Hz with excitation at 568 nm and collecting emission at > 560 nm, using a Zeiss Plan-Apochromat 63x/1.4 numerical aperture oil immersion objective, and the confocal pinhole was set to obtain spatial resolutions of 0.4 µm in the horizontal plane and 1 µm in the axial dimension. with an LSM-510 inverted confocal microscope (Carl Zeiss Inc., Jena, Germany). The occurrence of mPTP induction is clearly identified by the immediate dissipation of mitochondrial membrane potential (**Supplemental Figure 2**). ^37^ The ROS threshold for mPTP induction (tMPT) was measured as the average time necessary to induce mPTP in the laser exposed mitochondria. Images were processed by MetaMorph image analysis software (Molecular Devices, San Jose, CA).

***Autophagic Activity***

The cells were analyzed between 2- and 8-hours post-isolation and were taken from N = 3 rats. To measure autophagic activity cardiomyocytes were loaded with a vital dye from CYTO-ID Autophagy detection kit (Enzo Life Sciences, Inc., Farmingdale, NY; #51031-K200), according to the manufacturer protocol (dilution 1:500 in HEPES buffer) and incubated for 30 min at room temperature. The dye was washed out with HEPES buffer and cells imaged with excitation at 488 nm and collecting emission at > 505 nm, using 63x/1.4 NA oil objective with a Zeiss LSM-510 inverted confocal microscope (**Supplemental Figure 2**).

***Lipofuscin Accumulation***

To determine lipofuscin accumulation the cellular autofluorescence was imaged using 633 nm laser excitation and collecting emission at > 650 nm with 63x/1.4 NA oil objective on Zeiss LSM-510 confocal microscope. The cells were analyzed between 2 and 8 hours post-isolation and were taken from N = 6 rats.

Images of cardiomyocytes with stained autophagolysosomes and lipofuscin accumulation were analyzed by MetaMorph image analysis software (Molecular Devices), as described previously.^38^ The area of cell occupied by autophagolysosomes or lipofuscin was expressed as fraction of total cell area (**Supplemental Figure 2**). ^38^

***Frailty Index***

In brief, the frailty index was calculated by summing the number of deficits in factors related to integument, physical/musculoskeletal, vestibulocochlear/auditory, ocular/nasal, digestive/urogenital, respiratory, and aspects of physical discomfort for each rat and dividing by the total number of possible deficits. Along with these indices, rat weight and body temperature were also scored. A higher frailty index, with a potential score ranging from 0 to 1, is associated with lower survivability in rodents. ^32^

**Supplemental Figure 1 – Overall Study Design.** Red arrows representing natural death and blue arrows representing animals that were culled for analysis, with the number of rats below.

***
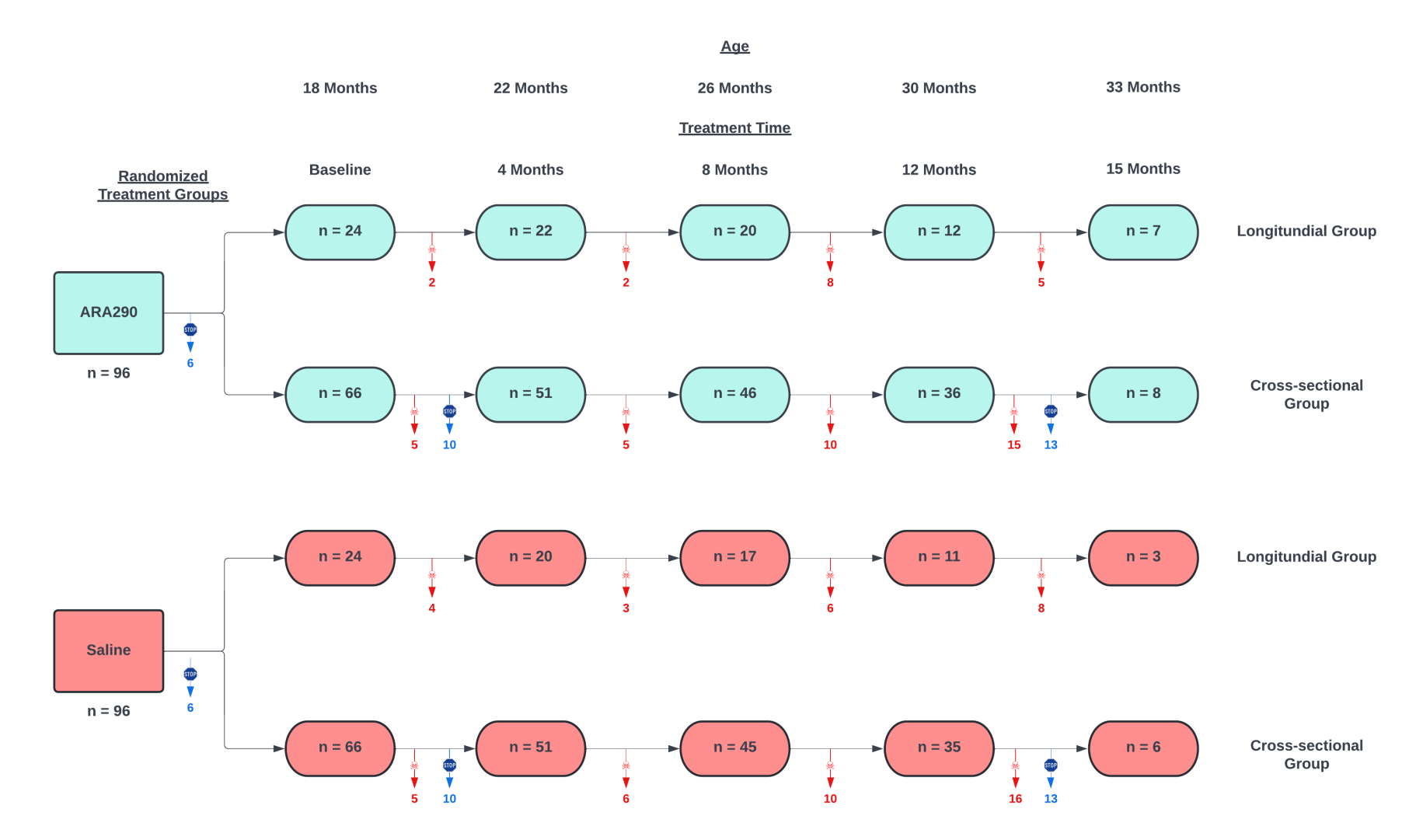
***

**Supplemental Figure 2 – mPTP, Autophagolysosome and Lipofuscin Experiments**


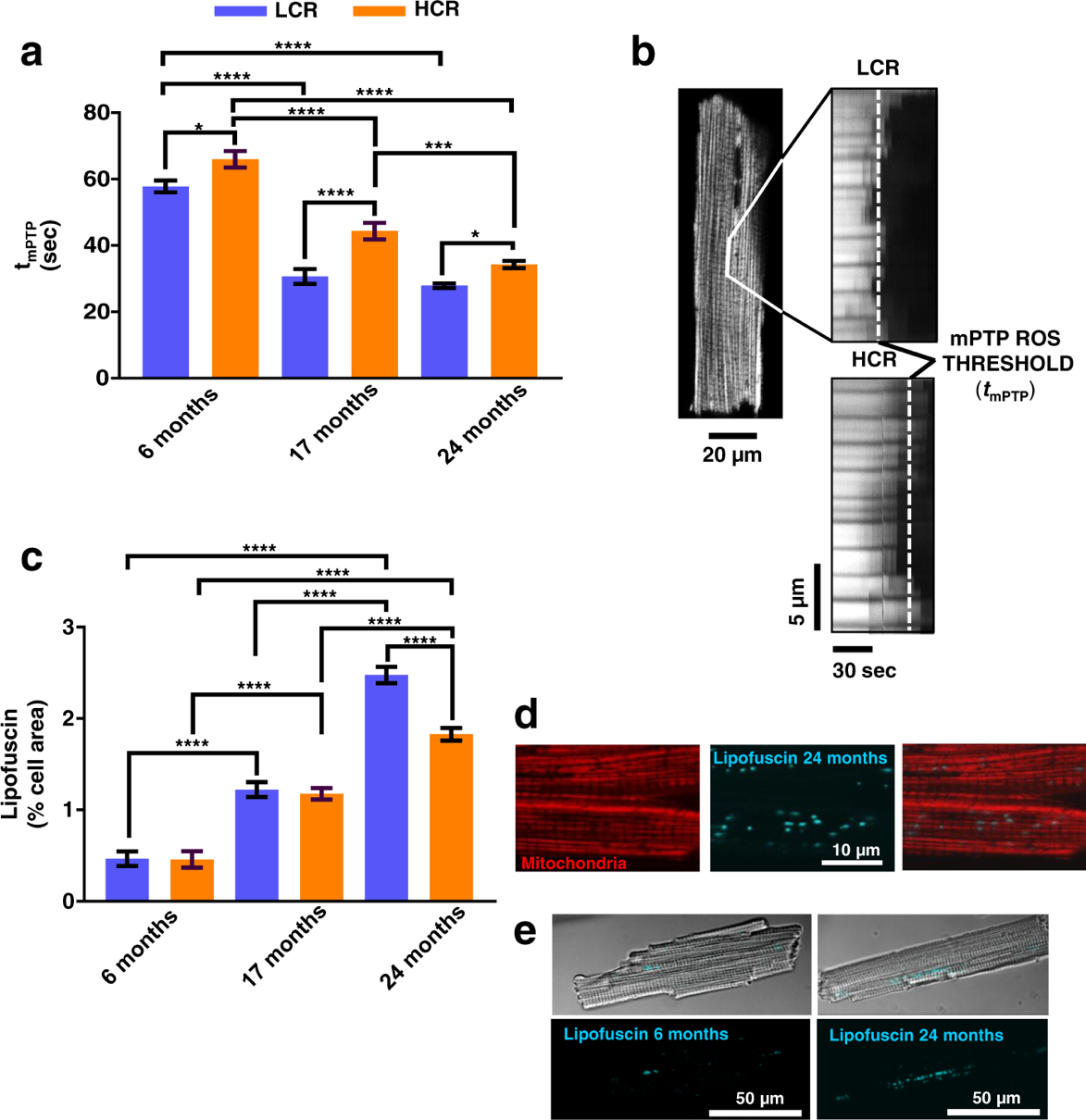
**
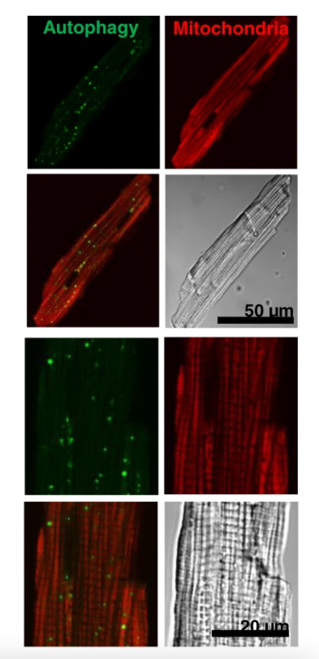
**

**
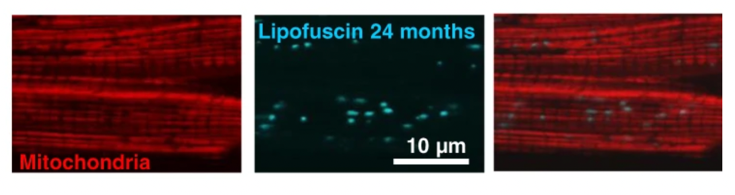
**

**Reference:** Aon, M.A., Cortassa, S., Juhaszova, M. *et al.* Mitochondrial health is enhanced in rats with higher vs. lower intrinsic exercise capacity and extended lifespan. *npj Aging Mech Dis* **7,**1 (2021). https://doi.org/10.1038/s41514-020-00054-3

**
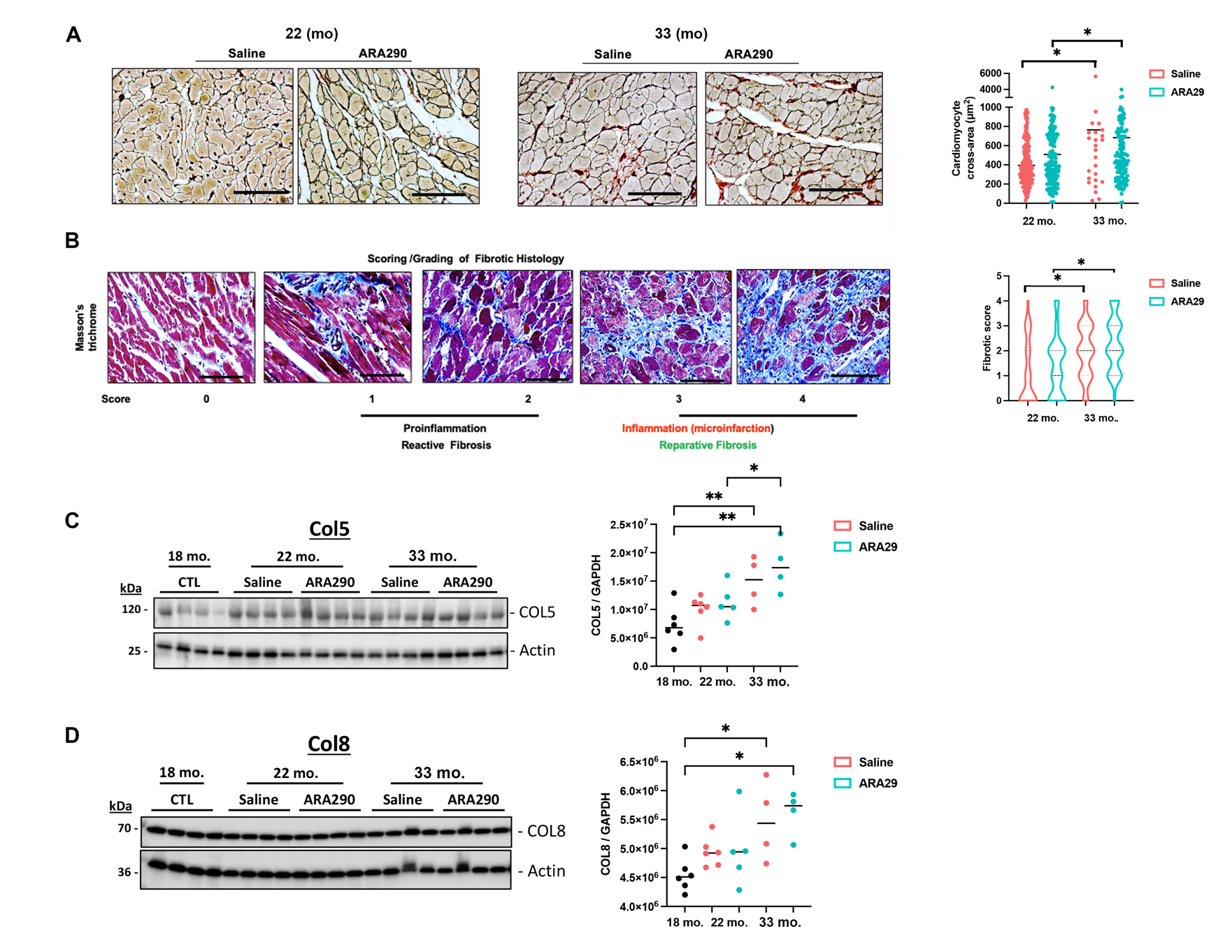
Supplemental Figure 3 – Histomorphometric Analysis**

**
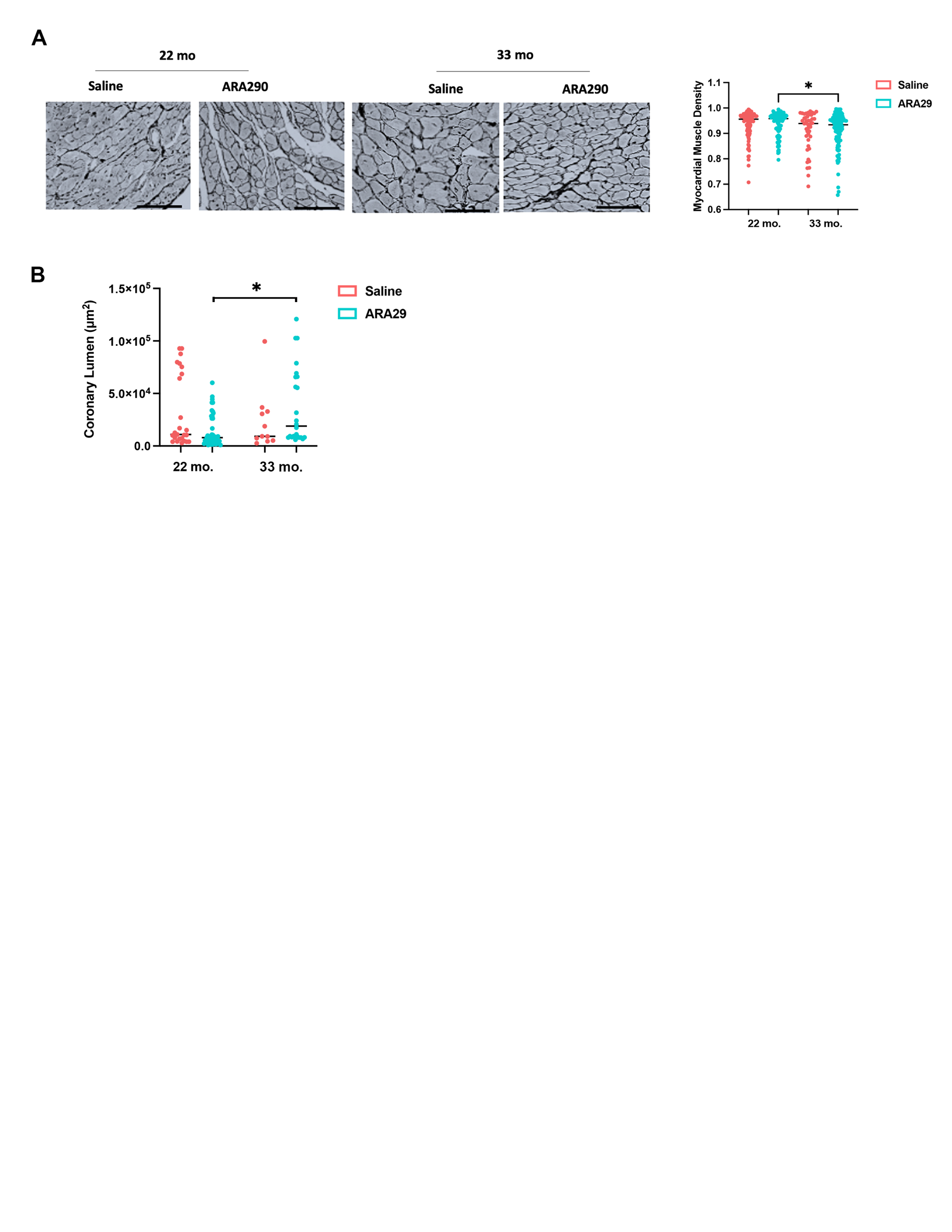
Supplemental Figure 4 – Histomorphometric Analysis**

**Supplemental Figure 5 – Kaplan-Meier Survival Curves**

**
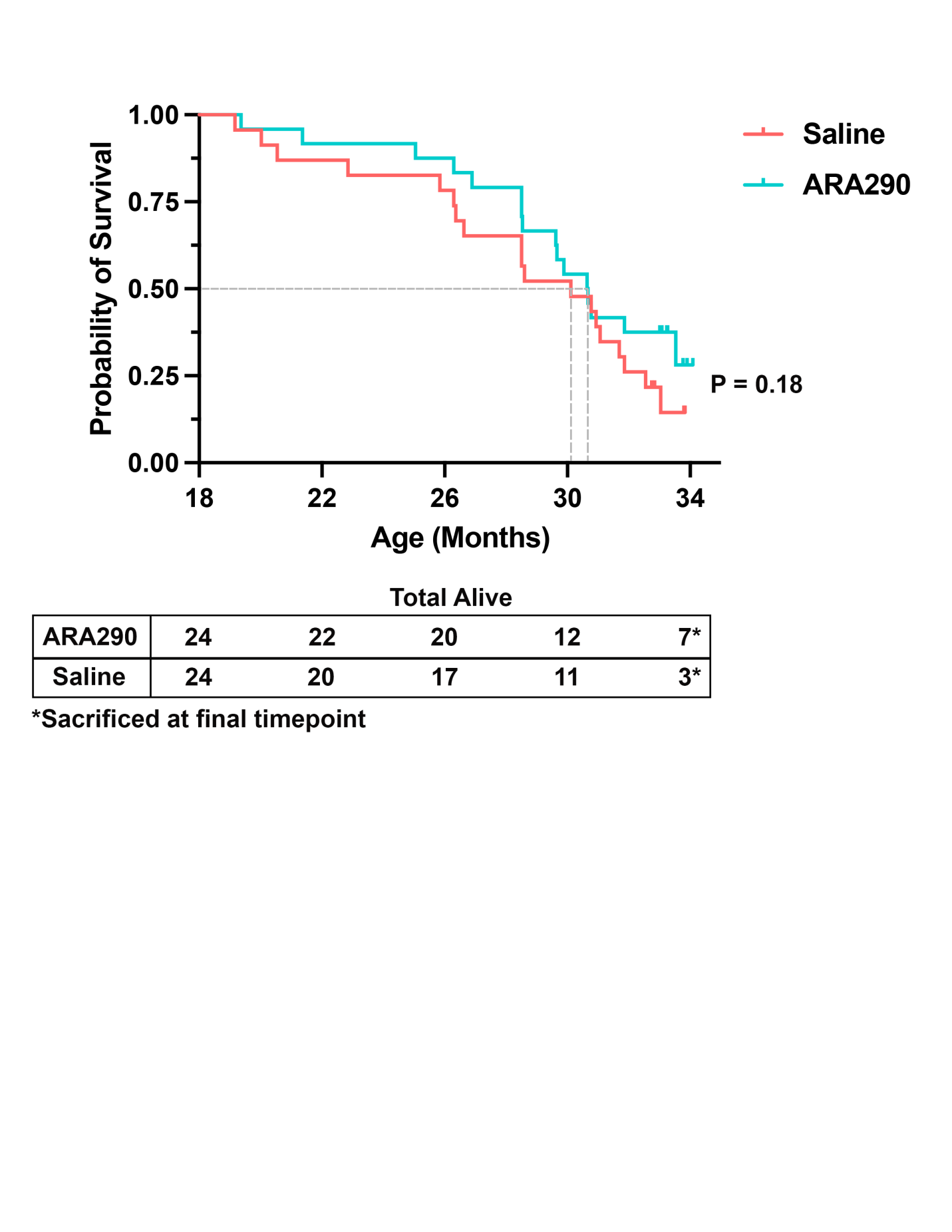
**

**Supplemental Table 1 – List of antibodies used for Western Blot Analyses**

| **Target Protein** | **Company** | **Catalog Number** | **Dilution** |
| --- | --- | --- | --- |
| Collagen Type V | ThermoFisher Scientific (Waltham, MA) | PA5-102416 | 1:1000 |
| Collagen Type VIII | ThermoFisher Scientific (Waltham, MA) | 17251-1-AP | 1:1000 |
| GAPDH | Cell Signaling Technology (Danvers, MA) | 2118 | 1:5000 |
| p-NFKb p65 Ser536 | ThermoFisher Scientific (Waltham, MA) | 44-711G | 1:1000 |
| NFKb p65 (RELA) | Cell Signaling Technology (Danvers, MA) | 8242 | 1:1000 |
| TNF alpha | Abcam (Cambridge, United Kingdom) | ab205587 | 1:1000 |

**Supplemental Table 2 – Mixed ANOVA Analysis of Age, ARA290 Treatment and Age-ARA290 Interaction**

| **Variable** | **Age Effects** | **ARA290 Treatment Effects** | **Age-Treatment Interaction** |
| --- | --- | --- | --- |
| **LVDd (cm)** | 0.01 | 0.04 | 0.33 |
| **LVDs (cm)** | 2.3E-12 | 0.0002 | 0.04 |
| **FS (%)** | 2.2E-16 | 5.4E-05 | 0.01 |
| **LVED (mL)** | 0.001 | 0.05 | 0.39 |
| **LVES (mL)** | 1.1E-12 | 0.0002 | 0.03 |
| **SV (mL)** | 0.24 | 0.33 | 0.81 |
| **EF (%)** | 3.6E-15 | 3.4E-05 | 0.01 |
| **HR (bpm)** | 9.7E-05 | 0.22 | 0.07 |
| **CO (mL/min)** | 0.43 | 0.27 | 0.53 |
| **CI (ml/min*g)** | 0.61 | 0.13 | 0.49 |
| **LVPWTHd (cm)** | 0.50 | 0.79 | 0.62 |
| **IVSTd (cm)** | 3.3E-05 | 0.02 | 0.34 |
| **LVMass (g)** | 0.40 | 0.44 | 0.86 |
| **LVPWTHs (cm)** | 4.1E-07 | 0.28 | 0.16 |
| **% PW Thickening** | 0.008 | 0.20 | 0.59 |
| **IVSTs (cm)** | 0.006 | 0.12 | 0.20 |
| **% IVS Thickening** | 0.34 | 0.73 | 0.83 |
| **PWV (cm/s)** | 0.14 | 0.70 | 0.49 |
| **BP (mmHg)** | 0.01 | 0.04 | 0.34 |
| **BW (g)** | 2.7E-14 | 0.07 | 0.19 |

**Left Ventricular Diameter (end diastole), LVDd; Left Ventricular Diameter (end systole), LVDs; Fractional Shortening, FS; Left Ventricular Volume (end diastole), LVED; Left Ventricular Volume (end systole), LVES; Stroke Volume, SV; Ejection Fraction, EF; Heart Rate, HR; Cardiac Output, CO; Cardiac Index, CI; Left Ventricular Posterior Wall Thickness (end diastole), LVPWTHd; Interventricular Septum Thickness (end diastole), IVSd; Left Ventricular Mass, LVM; Left Ventricular Posterior Wall Thickness (end systole), LVPWTHs; Percentage Left Ventricular Posterior Wall Thickening, % PW Thickening; Interventricular Septum Thickness (end systole), IVSTs; Percentage Left Ventricular Interventricular Septum Thickening, % IVS Thickening; Pulse Wave Velocity, PWV; Blood Pressure, BP; Body Weight, BW.**

**Supplemental Table 3, Page 1– Descriptive Statistics**

**Left Ventricular Diameter (end diastole), LVDd; Left Ventricular Diameter (end systole), LVDs; Fractional Shortening, FS; Left Ventricular Volume (end diastole), LVED; Left Ventricular Volume (end systole), LVES; Stroke Volume, SV; Ejection Fraction, EF; Heart Rate, HR; Cardiac Output, CO; Cardiac Index, CI; Left Ventricular Posterior Wall Thickness (end diastole), LVPWTHd; Interventricular Septum Thickness (end diastole), IVSd; Left Ventricular Mass, LVM; Left Ventricular Posterior Wall Thickness (end systole), LVPWTHs; Percentage Left Ventricular Posterior Wall Thickening, % PW Thickening; Interventricular Septum Thickness (end systole), IVSs; Percentage Left Ventricular Interventricular Septum Thickening, % IVS Thickening; Pulse Wave Velocity, PWV; Blood Pressure, BP; Body Weight, BW.**

**Supplemental Table 3, Page 2– Descriptive Statistics**

**Left Ventricular Diameter (end diastole), LVDd; Left Ventricular Diameter (end systole), LVDs; Fractional Shortening, FS; Left Ventricular Volume (end diastole), LVED; Left Ventricular Volume (end systole), LVES; Stroke Volume, SV; Ejection Fraction, EF; Heart Rate, HR; Cardiac Output, CO; Cardiac Index, CI; Left Ventricular Posterior Wall Thickness (end diastole), LVPWTHd; Interventricular Septum Thickness (end diastole), IVSd; Left Ventricular Mass, LVM; Left Ventricular Posterior Wall Thickness (end systole), LVPWTHs; Percentage Left Ventricular Posterior Wall Thickening, % PW Thickening; Interventricular Septum Thickness (end systole), IVSs; Percentage Left Ventricular Interventricular Septum Thickening, % IVS Thickening; Pulse Wave Velocity, PWV; Blood Pressure, BP; Body Weight, BW; *Sacrificed at the final timepoint.**
